# Notes and News

**Published:** 1903-10-24

**Authors:** 


### NOTES AND NEWS.

Her Royal Highness the Princess of Wales has become patron of the British Lying-in Hospital, which was founded in 1749.

An action has been brought against the Metropolitan Asylums Board for the alleged premature discharge of a patient which was followed by the spread of scarlet fever in his family.

Yellow Fever is very prevalent at present in Mexico, and it is reported to have spread to Texas. The inhabitants of the infected districts in Mexico are fleeing from the country, only to be met, if discovered, with a threat of being shot if they do not turn back.

The late Mr. Charles Samuel has bequeathed £500 each to St. Mary's, the Metropolitan, the Royal Free Hospital, the Mount Vernon Hospital for Consumption, and the City of London Hospital for Diseases of the Chest.

The *Times* publishes an appeal by Dr. Pagenstecher on behalf of the Wiesbaden Eye Hospital, which, he says, has received many poor English patients. We feel sure it does not need any reminder of the kind to stimulate the sympathy and generosity of the English people towards an institution where so world-wide a benefactor as Dr. Pagenstecher carries on his useful work, and we hope that the many who have benefited directly and indirectly from the great eye specialist's ministrations will enable him to build such a hospital as he needs.

It being held that a large amount of the nervous disorders of the circulatory system prevalent in Austria are due to the excessive use of tobacco, two new sorts of cigars have been introduced containing the very smallest percentage of nicotine. The nicotine is removed from the tobacco by washing the leaves of the plant. The sale of tobacco in Austria is a government monopoly, and therefore any organised experiment of this kind can be carried out systematically. We have not learnt whether the tobacco so treated is less popular than that with the full complement of nicotine.

The annual meeting of the Therapeutical Society will be held on October 27th at the Apothecaries' Hall, at 4 p.m.

One of the first resolutions passed by the Court of the new Victoria University of Manchester was one giving representation on the governing body of the university to the Royal Infirmary and other important institutions.

A general meeting of the Governors of St. Bartholomew's Hospital, to consider the report of the Lord Mayor's Committee on the Improvement Scheme for the Hospital, will be held on November 5th.

The Marseilles sanitary authority is making a most determined effort to exterminate rats. The Pasteur Institute supplies dansyg, a virus peculiarly fatal to rats, and as many as a thousand bodies are reported to have been counted in the docks, railway stations and other places in one night.

The coming winter course of lectures at the Brompton Consumption Hospital opened with the first two lectures by Dr. Habershon on "Bronchiectasis," which are open to members of the medical profession. The lectures will be held on October 21st and 28th at 4 p.m.

The entry of students at the London School of Tropical Medicine is the largest on record. About thirty post-graduates have already joined for the Autumn Session, nearly half being sent from the Colonial Office, Foreign Office, and Indian Medical Service.

The Metropolitan Asylums Board has arranged to allow free conveyance in their ambulances for patients suffering from measles where they are not in a position to pay. Previously hospitals have often been placed in the position of having to pay for the removal of a poor patient themselves.

A tropical veterinary department has been established in connection with the Liverpool Institute of Comparative Pathology. The object of this establishment is to train veterinary and medical men in the tropical diseases of animals, and to promote research and the prevention of disease. Liverpool is becoming so important a centre of all information relative to tropical medicine that this latest addition to its resources is well chosen and likely to be made very useful.

The mystery attending the disappearance of Miss Sophia Hickman, M.D., has been solved in a melancholy manner; her body was found last Sunday in Sidmouth Wood, Richmond Park, by some boys who were trespassing. The body was much decomposed, and had apparently remained in the spot where it was found since the date of her disappearance two months ago. Her watch and other valuables were found upon her. Soon after Miss Hickman's disappearance from the Royal Free Hospital her father wrote to the press remarking upon his daughter's fondness for the locality in which her body has been found. A reward of £200 was offered for her discovery by Mr. Hickman and the hospital authorities.

